# CD226: An Emerging Role in Immunologic Diseases

**DOI:** 10.3389/fcell.2020.00564

**Published:** 2020-07-24

**Authors:** Zhiyi Huang, Guangyin Qi, Joseph S. Miller, Song Guo Zheng

**Affiliations:** ^1^Laboratory of Tumor Immunology and Microenvironmental Regulation, Guilin Medical University, Guilin, China; ^2^Ohio University Heritage College of Osteopathic Medicine, Dublin, OH, United States; ^3^Department of Internal Medicine, The Ohio State University College of Medicine and Wexner Medical Center, Columbus, OH, United States

**Keywords:** CD226, adhesion factor, immune, autoimmune disease, tumor

## Abstract

CD226, a member of the immunoglobulin superfamily, is a functional protein initially expressed on natural killer and T cells. In recent years, the function of CD226 has been increasingly realized and researched. Accumulating evidence shows that CD226 is closely related to the occurrence of autoimmune diseases, infectious diseases, and tumors. Because of the CD226’s increasing importance, the author herein discusses the structure, mechanism of action, and role of CD226 in various pathophysiological environments, allowing for further understanding of the function of CD226 and providing the basis for further research in related diseases.

## The Structure of CD226

### Discovery and Naming

CD226, namely T lineage specific activation antigen 1 (TLisA1), platelet and T cell antigen 1 (PTA-1), or DNAM-1, is a member of the immunoglobulin superfamily. The molecular membrane contains two V-like domains of immunoglobulin (Shibuya et al., 1996; Sherrington et al., 1997). Burns et al. first found that CD226 was expressed on the surface of T cells in 1985, documenting that it was related to the activation of CTL, so it was named TLisA1 (Burns et al., 1986). Later studies revealed that CD226 was also expressed in platelets and involved in platelet activation aggregation, so it was called PTA1 (Shibuya et al., 1996). In 1997, Burns et al. confirmed that TLisA1 and DNAM-1 are actually the same molecule (Sherrington et al., 1997). In 2000, at the Seventh International Conference of human leukocyte differentiation antigen collaboration group, the molecule was officially named CD226 (Mason et al., 2001).

### Gene and Structure

**FIGURE 1:**
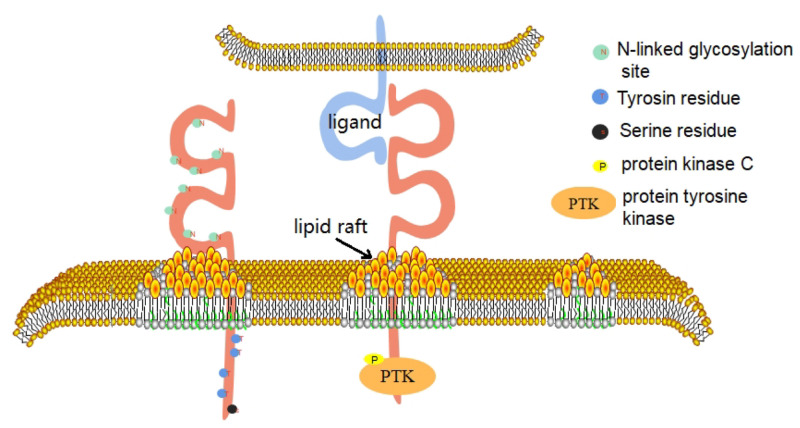
CD226 consists of three domains. The extracellular domain includes 2 immunoglobulin V-like domains and 8 N-linked glycoprotein sites. The first domain outside the envelope of CD226 molecule is its structural basis for recognition of ligands, adhesion, immune synapse formation and cytotoxic effect. The intracellular domain contains 4 tyrosine residues and 1 serine residue. When CD226 binds to its ligand, the CD226 molecule directionally moves to lipid rafts on the cell membrane and recruits intracellular signaling molecules such as PTK and PKC, which phosphorylate on the four residues and activate the cell.

CD226 is a conserved sequence in human and mouse genes, which are located in the 18q22.3 and 18E4 bands of the chromosome, respectively (Jinlong et al., 2002). In 2002, the CD226 gene of mice was successfully identified, which has a total length of 2,487 BP. It contains an open reading frame of 1002 BP and encodes a leading sequence of 18 amino acids and a mature CD226 protein of 315 amino acids (Xinhai et al., 2002). In 2006, the promoter sequence of human CD226 gene was identified (Jian et al., 2006). The gene has at least two promoters, which are located at −810 to −287 bp and +33 to +213 bp, that have distinct tissue-specific roles and are physically separated by a negative regulatory element (Jian et al., 2006). Human CD226 gene contains 7 exons and 6 introns, of which exon 7 encodes 41 amino acids in the cytoplasmic region (Jinlong et al., 2002). Bioinformatics analysis shows that, within this CD226 gene region, there are putative binding sites for transcription factors AP-1, Sp1, PEA3, and Ets-1 (Jian et al., 2006). The full length of human CD226 cDNA is 2,664 BP, which contains an open reading frame and encodes a leading sequence of 18 amino acids and a mature CD226 protein of 318 amino acids. The extracellular domain contains 230 amino acids, including 2 immunoglobulin V-like domains, and 8 N-linked glycosylation sites and it is thus extremely susceptible to degradation of its glycan residues; deglycosylation yields a 35 kDa protein (Shibuya et al., 1996). The transmembrane domain contains 28 amino acids. The intracellular domain contains 60 amino acids. The intracellular domain contains 4 tyrosine residues and 1 serine residue, which can collect signal proteins after phosphorylation. This reaction is based on the interaction between CD226 and its ligand (Jun et al., 2012). The first domain outside the envelope of CD226 molecule is its structural basis for recognition of ligands, adhesion, immune synapse formation, and cytotoxic effect (Shengke et al., 2014; Figure 1).

There are three kinds of single nucleotide mutations in the exon of CD226 gene, including CD226 rs763361, rs34794968, and rs727088 (Bossini-Castillo et al., 2012). These three single nucleotide polymorphisms of CD226 gene have been confirmed to be related to the susceptibility of a variety of autoimmune diseases (Bossini-Castillo et al., 2012). CD226 rs763361/gly307ser non-synonymous mutations are associated with susceptibility to autoimmune diseases such as type 1 diabetes (T1D), rheumatoid arthritis (RA), multiple sclerosis (MS), autoimmune thyroid disease (AITD), and systemic sclerosis (SSc; Todd et al., 2007; Qiu et al., 2008; Smyth et al., 2008; Dieudé et al., 2011). Because the non-synonymous mutation encoded by this allele can encode the cytoplasmic tail (exon 7) of CD226 protein, this mutation may affect the function of T cells or other cells (Todd et al., 2007). Indeed, T cell and other cell dysfunction is closely associated with the onset and development of autoimmune diseases (Li et al., 2013; Maogen et al., 2014; Yaoyao et al., 2018). Other hypotheses suggest that this mutation may disrupt the binding sites of enhancers and/or silencers, thereby changing RNA splicing (Todd et al., 2007). In addition, the replacement of rs763361 with serine may also interfere with the phosphorylation of 322 tyrosine and 329 serine sites of CD226, as well as post-translational modifications in downstream signaling pathways (Todd et al., 2007; Boquan and Zhuwei, 2010). CD226 rs727088, a single nucleotide polymorphism, may influenc the expression of CD226 at the transcription level, which has been found to be highly correlated with tumor susceptibility (Löfgren et al., 2010). The single nucleotide mutation of CD226 rs34794968 alone does not affect the occurrence and development of the disease, but it can play a synergistic role with the above two mutations (Bossini-Castillo et al., 2012).

### Expression and Distribution

The expression patterns of CD226 are varied (Ralston et al., 2004). In the peripheral blood, CD226 is expressed on T cells, NK cells, NK T cells, B cells, monocyte/macrophage, dendritic cells (DC), megakaryocyte/platelet lineage, and hematopoietic precursor cells (Burns et al., 1986; Scott et al., 1989; Kojima et al., 2003; Shibuya et al., 2003; Reymond et al., 2004; Dongchu et al., 2005). Endothelial cells also display low quantities of this protein in resting conditions, yet expression is significantly enhanced upon their stimulation (Lihua et al., 2003). CD226 is also expressed on mature mast cell and on bone marrow-derived CD34 + progenitor cells, but not on progenitors of the erythrocytic lineage (Dongchu et al., 2005; Bachelet et al., 2006). It was demonstrated that CD226 + NK cells play an important role in the recognition of several types of human tumors, such as myeloma, melanoma, and ovarian carcinoma, and Recent studies have shown that CD226 may be one of the markers of mature NK cells (Martinet et al., 2015). We previously reported that CD226 + NK cells are elevated in lupus and predominately infiltrated the lupus kidney. In addition, these activated NK cells mediated tissue injury by producing cytotoxic granules, eventually contributing to lupus nephritis (Huang et al., 2011).

### Ligands of CD226

In 2003, Bottino et al. confirmed that the ligand of human CD226 was CD155 (nacl-5, PVR) and CD112 (nicotine-2) which have a similar molecular weight, 70 kD, and 65/60 KD. They belong to the members of the nicotine-like protein family and the nicotine protein family (Bottino et al., 2003; Wang et al., 2009). In 2005, Tahara-Hanaoka et al. (2005) demonstrated that the ligands of mouse CD226 molecules are also CD155 and CD112. CD155 and CD112 molecules are widely expressed in a variety of tissue cells, such as nerve cells, endothelial cells, epithelial cells, antigen-presenting cells, fibroblasts, pathogen infected cells, and a variety of tumor cells (Bottino et al., 2003; Tahara-Hanaoka et al., 2004; Dardalhon et al., 2005; Bryceson et al., 2006; Pende et al., 2006; Gilfillan et al., 2008; Kraus et al., 2016). The solid cancers, as well as hematological malignancies, have high levels of CD155 and CD112 making them good targets for CTL attack via CD226-specific binding (Castriconi et al., 2004; Pende et al., 2005; Moretta et al., 2006; Carlsten et al., 2007; El-Sherbiny et al., 2007). They are also expressed in immune cells such as monocytes, DCs, and activated T cells (Pende et al., 2006), and also affect some physiological processes.

## The Function of CD226

### CD226 Participates in the Function of CTL Cells and NK Cells

In human body, CD226 is highly expressed on the surface of NK cells and CD8 + T cells (Shibuya et al., 1996). In mice, 40–50% of NK cells and all CTL cells constitutively express CD226 (Dardalhon et al., 2005).

CD226, as an adhesion molecule, promotes the migration, activation, proliferation, differentiation, and function of CD8 + T cells. CTL cells need to migrate to the inflammatory site or tumor microenvironment through adhesion molecules to establish close contact (Bryceson et al., 2006). In the secondary lymphoid organs, adhesion molecules mediate the interaction between CTL cells and antigen-presenting cells, and finally activate, proliferate and differentiate them. The interaction of CD226/CD155 is very important for the proliferation of CD8 + T cells and the immune response of antigen-specific CD8 + T cells (Gilfillan et al., 2008). The co-stimulatory signaling pathway of CD8 + T cells mediated by CD226 was interrupted in the process of chronic HIV-1 infection. Therefore, the expression of CD226 on the surface of CD8 + T cells is down regulated, which impairs the effect of CTL (Cella et al., 2010).

More and more evidences show that CD226 is involved in the biological function of NK cells (Enqvist et al., 2015; Martinet and Smyth, 2015; Martinet et al., 2015). The combination of CD226 with CD155 and/or CD112, in cooperation with NKp30, can induce NK cells to dissolve immature DC and promote the proliferation of mature DC (Balsamo et al., 2009). DC maturation promotes immune response by enhancing adaptive immune system (Zheng et al., 2006; Ramalingam et al., 2012; Peng et al., 2020). The interaction between CD226 and its ligands is involved in the cross-linking of NK and T cells. In graft versus host disease (GVHD; Lozano et al., 2013) and other autoimmune diseases (Ardolino et al., 2011), NK cells can recognize and kill antigen stimulated T cells that have been activated/proliferated and can also promote the differentiation of helper T cells.

CD226, together with CD96, TIGIT, and CRTAM, is also involved in the regulation of NK cell function (Ralston et al., 2004; Chan et al., 2014; Nabekura et al., 2014; Shu-Bin et al., 2020). After LPS stimulation, the proportion of IFN-γ + NK cells in CD226 deficient mice was significantly lower than that in wild-type mice (Chan et al., 2014). CD96 is believed to inhibit the secretion of IFN-γ by NK cells. Therefore, CD96 and CD226 molecules reverse the secretion of IFN-γ in NK cells (Chan et al., 2014). TIGIT, CD96, and CRTAM are able to recognize the nectin and Necl family molecules and regulate the function of NK cells, which further complicates the research of CD226 in the biological function of NK cells (Chan et al., 2014). In recent years, it has been reported that the synergistic effect of CD226 and these three molecules balances the activation of NK cells *in vivo* (Martinet and Smyth, 2015).

### CD226 Participates in the Function of CD4 + T Cells

In 2005, Dardalhon et al. (2005) stated that CD226 was a specific surface marker of Th1 cells in mice. In 2006, Shibuya et al. revealed that the freshly isolated CD4 + T cells in the mice also expressed low level CD226 molecules, that initial T cells expressed CD226 molecules, and that the most polarized Th1 and Th2 cells also expressed CD226 molecules (Shibuya et al., 2006). In 2012, Lozano et al. demonstrated that TIGIT can inhibit T cell functions by competing with CD226 (Lozano et al., 2012). In 2015, Fuhrman et al. found that CD226 was expressed in memory CD4 + T cells (Fuhrman et al., 2015). These abnormal cells and relative pro-inflammatory cytokines contribute to many immunologic diseases (Yang and Song Guo, 2016; Sujuan et al., 2018, 2019).

In 2013, Sutavani et al. (2013) proved that the surface marker of human type 1 regulatory T cells (Tr1) is CD4 + CD49b + LAG-3 + CD226+. Tr1 is a kind of chronic activated CD4 + T cells in the presence of IL-10 (Weishan et al., 2017; Fang et al., 2018), which has the functions of low proliferation, high secretion of IL-10, low expression of IL-2, and IL-4 (Gagliani et al., 2013). CD226 is highly expressed on the surface of human type I regulatory T cells and participates in the killing of myeloid antigen presenting cells (Magnani et al., 2011). Tr1 has the function of down regulating immune response and maintaining peripheral immune tolerance. It has the application prospect in the treatment of autoimmune diseases, tumors, and the rejection of allogeneic tissue and organ transplantation.

Recently, it has been found that CD226 and TIGIT molecules expressed on the surface of human CD4 + Foxp3 + Treg cells are related to their stability and inhibition. Similar to Tr1 cells, CD4 + Foxp3 + Treg are crucial for maintaining homeostasis and preventing autoimmune issues (Ya et al., 2014; Anping et al., 2016). The purity and inhibitory function of Treg subsets of CD226 + TIGIT- will be weakened after amplification, and with the increase of IL-10 and effector cytokines, it is suggested that the expression of CD226 affects the function of Treg (Fuhrman et al., 2015). Foxp3, Helios were highly expressed in CD226-TIGIT + Treg cells and with Treg-specific demethylated region. *In vitro* inhibition experiments show that TIGIT expression in Treg cells is related to its strong inhibitory activity. On the other hand, the expression level of CD226 in activated Treg cells is up-regulated. Therefore, in the process of looking for blocking CD226 to weaken the activity of traditional effector T cells, attention should be focused on appropriate doses to avoid the simultaneous reduction of Treg function (Fuhrman et al., 2015). However, in patients with RA, we recently demonstrated that while CD226 and TIGIT both showed elevated expression levels in CD4 + Foxp3 + cells, they were not associated with disease activity of RA patients (Mengru et al., 2019). Thus, CD226 doesn’t seem to be an ideal marker for human Treg cells.

### CD226 Is Involved in the Function of Other Cells

CD226 is also expressed in platelets. The cross-linking of CD226 and mAb can induce platelet activation, allowing thrombin to induce CD226 tyrosine phosphorylation and mediate platelet adhesion (Scott et al., 1989). Subsequently, it is documented that CD226 mediates the adhesion of platelets and megakaryocytes to vascular endothelial cells (Kojima et al., 2003).

**FIGURE 2:**
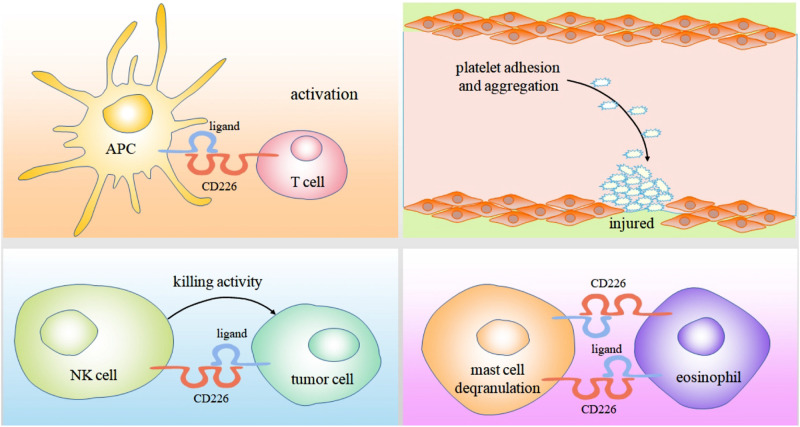
As an adhesion molecule, Binding of CD226 and CD155 can mediate the interaction between CTL cells and professional antigen presenting cells, ultimately enabling them to activate, proliferate and differentiate. CD226 also can induces platelet activation and mediates platelet adhesion by cross-linking with its mAb. The killing of tumor cells by NK cells mainly depends on the binding of CD226 to CD112 and CD155 expressed on tumor cells. In the presence of eosinophils, mast cell FCεRI mediated degranulation is enhanced. This effect is partly caused by the interaction of CD226/CD112.

Human mast cells and eosinophils can simultaneously express CD226 and CD112. These cells play an important role in promoting allergic diseases (Wenru et al., 2012, 2014, 2015). In late and chronic stages of anaphylactic inflammation, mast cells and eosinophils with tissue infiltration form a regulatory unit. In the presence of eosinophils, mast cell FCεRI mediated degranulation is enhanced. This effect is partly caused by the interaction of CD226/CD112 triggering Fyn, LAT, and phospholipase Cγ2 signaling pathways involved in the above progress (Bachelet et al., 2006) (Figure 2).

## CD226 and Clinical Diseases

### CD226 and Autoimmune Diseases

Previous studies indicated that the expression of CD226 is negatively correlated with the inhibitory function of Foxp3 + Tregs. TIGIT, a co-inhibitory molecule on T cells, exerts immunosuppressive effects by competing with CD226 for the same CD155 ligand (Lozano et al., 2012). TIGIT and CD226 costimulatory axis plays an important role in the immunoregulatory function of Foxp3 + Tregs and is related to several autoimmune diseases. Several lines of evidence support that the suppressive capacity of CD226 + Tregs is inhibited (Ning et al., 2019), and that TIGIT + Tregs are highly suppressive and more so (Joller et al., 2014). Experimental autoimmune encephalomyelitis (EAE) contributes to a breakdown of self-tolerance and leads to Th17 cells infiltrating the central nervous system to mediate inflammation and neuronal injury (Rostami and Ciric, 2013). Zhang et al. reported that EAE susceptibility in mice treated with anti-CD226 pAb was markedly decreased via balancing the Th17/Treg ratio (Rong et al., 2016), and the enhanced suppressive capacity of Tregs during EAE is related to the absence of CD226 and the increased expression levels of TIGIT (Ning et al., 2019). Ulcerative colitis (UC) is a chronic inflammatory immune-related disease. Long et al. found that lack of CD226 expression on Foxp3 + Tregs play a positive role in the recovery of clinical remission from active stage in UC patients and TIGIT expression on CD226-Foxp3 + Tregs have a potential positive effect on the suppression of CD226-Foxp3 + Tregs (Yan et al., 2020).

In recent years, with the development of sequencing technology, the relationship between gene and disease is becoming more clear. The study of CD226 gene polymorphism and autoimmune disease susceptibility has entered a new stage.

CD226 rs763361 is 307 glycine replaced by serine. This mutation may be related to the development of a series of immune related diseases, such as SLE, SSc, T1D, and RA (Du et al., 2011; Avouac et al., 2013; Mattana et al., 2014; Elhai et al., 2015). SSc is a chronic autoimmune disease that affects connective tissue, characterized by dermal fibrosis and skin thickening. In the CD226-/- mice model, the characteristics of fibrosis were weakened when compared to the wild-type mice. It is possible that the expression of CD226 promotes the development of SSc (Avouac et al., 2013). In the *in vitro* model of RA, CD226, and CD226Ls were expressed in NK cells and fibroblast like synovitis (FLS) of RA patients, respectively, suggesting that RA-FLS cells can be recognized and killed by NK cells (Nielsen et al., 2014). Many studies have found that autoimmune diseases are related to the dysfunction of NK cells. It is speculated that the 307 glycine serine mutation of CD226 may cause the dysfunction of NK cells (Fogel et al., 2013). The activation of T cells requires the guanine nucleotide exchange factor VAV1 (Katzav et al., 1989). CD226 engagement triggered VAV1 activation through tyrosine phosphorylation and synergized with signaling through T cell receptor (TCR) to positively regulate cytokine production by CD4 + T cells. Moreover, co-engagement of the TCR, and CD226 rs763361 that is associated with autoimmunity further enhanced VAV1 activation and IL-17 production (Gaud et al., 2018). All of the above results suggest the feasibility of targeting CD226 in the treatment of autoimmune diseases.

### CD226 and Tumors

CD226 is expressed on the surface of NK cells and CD8 + T cells. As an important NK cell activating receptor, CD226 is widely involved in various immune responses. Studies have shown that CD226 plays an important role in the killing of tumor cells by NK cells (Verhoeven et al., 2008; Lakshmikanth et al., 2009). Formation of stable conjugates with tumor cells is essential for NK cells to exert tumor killing effects, and CD226 enables prolonged stable interaction between NK and tumor cells (Kim et al., 2017). CD155 and CD112 are two important ligands of CD226. CD112 is down regulated in tumor tissue. It can combine with CD226 on the surface of NK cells, thereby activating NK cells to kill tumor cells (Xiaojun et al., 2014). Similarly, the decrease of CD155 in liver cancer tissue can reduce the cytotoxic effect mediated by NK cells (Jiuyu et al., 2014). An experiment *in vivo* has demonstrated that CD226 mediates phosphorylation of FOXO1 and activates NK cells through interaction with CD155-expressing tumor cells (Xiangnan et al., 2018). Tumor immunotherapy offers promising outcomes in patients with tumor. Concepción et al. discovered the importance of the CD226/immunoglobulin-like receptor ratio of NK cells induced by licensing interactions as critical determinants for solid cancer immune surveillance, and provides predictive biomarkers for patient survival that may also improve the selection of donors for NK cells immunotherapy (Guillamón et al., 2018). It is generally believed that Tregs in tumors impede T cell responses to tumors (Naturally, 2005). Among the current tumor immunotherapy regimens targeting Tregs, the biggest problem is the lack of highly selective drugs for Tregs. This is mainly due to the fact that highly specific markers have not yet been found in Tregs. Julien et al. indicated that a high TIGIT/CD226 ratio in Tregs regulates their suppressive function and stability in melanoma, and they suggested novel immunotherapies to activate CD226 in Tregs together with TIGIT blockade to counteract Treg suppression in cancer patients (Fourcade et al., 2018). In recent years, some researches began to pay attention to the expression of CD226 in tumor cells. These studies found that expression of CD226 on hepatoma cells was down-regulated and that expression was related to the survival rate and survival time of patients (Baoxing et al., 2017). In addition, CD226 gene polymorphism was found to be directly related to tumor risk (Shaoqing et al., 2013). All of the above results suggest CD226 may have anti-tumor effects. Thus, the role and mechanism of CD226 in various cancers warrants an in-depth study in the future.

### CD226 and Viral Infections

In most virus infected diseases, NK cells can eliminate virus-infected and transformed cells (Cooper et al., 2001; Smyth et al., 2005). They recognize target cells via a germ-line–encoded repertoire of activating and inhibitory receptors (Bryceson et al., 2006). CD226 is an important activating receptors. NK cells require CD226 for recognition of HCV-infected hepatoma cells (Stegmann et al., 2012) and HCMV-infected myeloid DCs (Magri et al., 2011). Human immunodeficiency virus (HIV) infection causes dysfunction of the innate and adaptive immune systems, and disturbs NK cells and CD8 + T cells function and the surface expression levels of some receptors (Watzl et al., 2014; Zhuo et al., 2017). Yin et al. (2018) demonstrates that TIGIT expression is specifically elevated on CD226 + NK cells in HIV-infected individuals, and high levels of TIGIT can inhibit IFN-γ production by NK cells, while blockade of TIGIT can restore their function. Similar results were found on CD8 + T cells in HIV-infected individuals. HIV-specific CD8 + T cells were almost exclusively TIGIT+, and HIV-specific TIGIT^hi^ cells were negatively correlated with polyfunctionality and displayed a diminished expression of CD226 (Tauriainen et al., 2017). All these highlight the important role of TIGIT/CD226 axis in viral infections and suggest a potential new avenue for the development of therapeutic strategies toward a functional cure.

## Conclusion

Since its discovery, CD226 has been demonstrated to be widely expressed on various immune cells and plays an important functional role in the immune system. Many aspects of the pathophysiological state of CD226 have not yet been clarified; however, current research on the function and mechanisms of this molecule is allowing for increased understanding of the molecule’s clinical relevance. Indeed, CD226, as a costimulatory factor, plays an important role in the development of various diseases. Thus, manipulation of CD226 expression and function may be a feasible therapeutic strategy for many immune related diseases and tumors.

## Data Availability Statement

The original contributions presented in the study are included in the article/supplementary material, further inquiries can be directed to the corresponding author.

## Author Contributions

ZH and GQ designed the study and wrote the review. SZ conceived and designed the review. SZ and JM edited and revised review. All authors discussed and approved the final version.

## Conflict of Interest

The authors declare that the research was conducted in the absence of any commercial or financial relationships that could be construed as a potential conflict of interest.
